# Complete plastid genome sequences of *Coreanomecon hylomeconoides* Nakai (Papaveraceae), a Korea endemic genus

**DOI:** 10.1080/23802359.2016.1209089

**Published:** 2016-09-05

**Authors:** Hoe-Won Kim, Ki-Joong Kim

**Affiliations:** Division of Life Sciences, Korea University, Seoul, Korea

**Keywords:** *Coreanomecon hylomeconoides*, Korean endemic genus, plastid genome

## Abstract

The plastid genome (plastome) sequences from the monotypic endemic genus of Korea, *Coreanomecon hylomeconoides* Nakai, were completed in this study (NCBI No. KT274030). The complete plastome size was 158,824 bp in length, containing a pair of 26,685 bp inverted repeat (IR), which were separated by a large and small single copy (LSC, SSC) region of 86,916 bp and 18,538 bp, respectively. The overall GC contents were 38.8%. The plastome contained 112 genes, including protein-coding genes, 30 tRNA genes, 4 rRNA genes. Sixteen genes contained one intron and two genes had two introns. Phylogenetic analysis revealed that *C. hylomeconoides* was sister group of *Papaver somniferum* with 100% support value.

*Coreanomecon hylomeconoides* Nakai (Papaveraceae) are monotypic endemic genus in Korea (Nakai 1935). It is a perenial herb that mainly distributed in the southern region of Korea. The genus *Coreanomecon* belongs to the subfamily Chelidonioideae which consists of 10 genera (Blattner & Kadereit 1999). In Korea, this subfamily comprised of three genera (*Chelidonium, Hylomecon, Coreanomecon*). Some authors combined *Coreanomecon* into *Hylomecon* or *Chelidonium* based on morphological characters (Ohwi 1953; Lee 1973). However, palogenetic study and cladistic analysis have supported that *Coreanomecon* is the distinctive Korea endemic genus (Lee & Kim 1984; Park et al. 1999). In this paper, we report the complete plastome sequence of *C. hylomeconoides.* This data will provide better information for plastome evolution and phylogenetic relationships for the Papaveraceae.

Materials were collected from the natural habitat of the Mt. Huia, Suncheon, Korea. Chloroplast DNA was isolated from approximately 100g fresh leaves using a sucrose gradient centrifugation method (Jansen et al. 2005). Sequencing was performed by Genome Sequencer FLX System (Roche, Basal, Switzerland), and assembled by GS De Novo Assembler (Roche Diagnostics Company). Junctions between the IR and the LSC and SSC confirmed using PCR and Sanger sequencing. Annotations was performed using the BLAST of the National Center for Biotechnology Information (NCBI), DOGMA (Wyman et al. 2004) and tRNAscan-SE (Lowe & Eddy 1997). The DNAs and voucher specimens used in this study were deposited in the Plant DNA Bank of Korea (PDBK 2008-0361) and Korea University Herbarium (KUS 2008-0361), respectively.

The plastome structure, gene order and content of *C. hylomeconoides* were similar to the typical land plant plastome (Palmer 1991). The *C. hylomeconoides* plastome contains a pair of inverted repeat regions (IRa and IRb) consisting of 26,685 bp each. The two IR regions were divided into LSC region of 86,916 and SSC region of 18,538 bp. The total cp genome is 158,824 bp in length. The plastome comprised 130 genes (113 unique), including 79 protein-coding genes, 30 tRNA genes, 4 rRNA genes. The overall GC contents of the plastome were 38.8%, and in the LSC, SSC and IR regions were 37.3%, 32.8%, 43.2%. A total of 18 genes contain one or two introns. Two genes, *ycf3* and *clpP* have two introns. The *rps12* gene is the unique divided gene in which the 5′ exon is located in the LSC region and two 3′ exons and introns are duplicated and located in the IR region.

To validate the phylogenetic relationships of *C. hylomeconoides* among Ranunculales, we constructed Maximum likelihood (ML) tree. Phylogenetic analyses were performed on a data set that included 79 protein-coding genes and four rRNA genes for 33 taxa using RAxML v. 8.2.8 on CIPRES (http://www.phylo.org). The 83 gene sequences (80,565 bp) were aligned with MUSCLE in Geneious v. 6.1.7 (Biomatters Ltd., Auckland, New Zealand). This analysis showed that Eupteleaceae was split first in Ranunculales followed by Papaveraceae (Figure 1). Therefore, the Eupteleaceae and Papaveraceae are the two early diverging lineages in the Ranunculales. Only two complete plastome sequences of *C. hylomeconoides* and *Papaver somniferum* are available from the Papaveraceae at this time. Two species form a sister group in the tree with 100% support value. In order to evaluate the validity of the endemic genus of the *Coreanomecom,* we need more comprehensive plastome data in the subfamily Chelidonioideae of the Papaveraceae.

**Figure 1.**
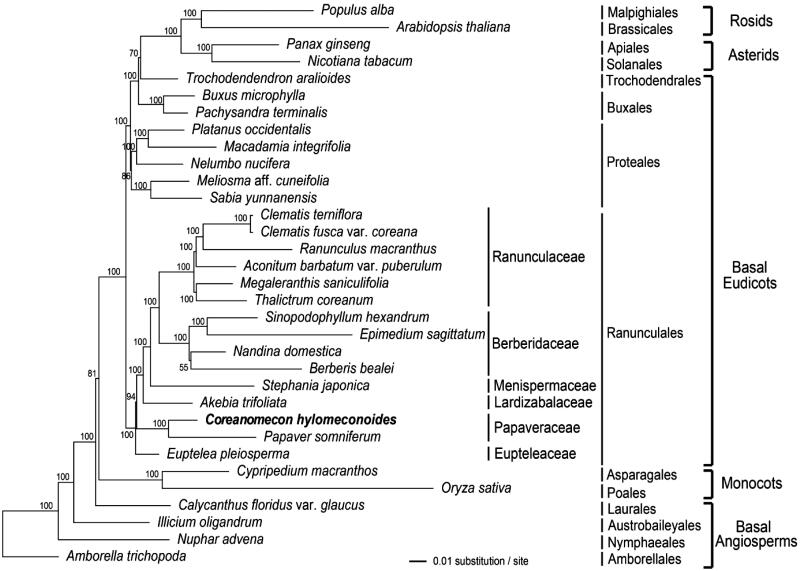
Maximum likelihood (ML) tree based on 79 protein-coding and four rRNA genes from 33 plastid genomes as determined by RAxML (-ln *L =* -545811.818157). The numbers at each node indicate the ML bootstrap values. Genbank accession numbers of taxa are shown below, *Aconitum barbatum* var. *puberulum* (KC844054), *Akebia trifoliata* (NC_029427), *Amborella trichopoda* (NC_005086), *Arabidopsis thaliana* (NC_000932), *Berberis bealei* (NC_022457), *Buxus microphylla* (NC_009599), *Calycanthus floridus* var. *glaucus* (NC_004993), *Clematis* fusca *var. coreana* (KM652489), *Clematis terniflora* (NC028000), *Coreanomecon hylomeconoides* (KT274030, in this study), *Cypripedium macranthos*(NC_024421)*, Epimedium sagitatum* (NC_029428), *Eutelea pleiosperma* (NC029429), *Illicium oligandrum* (NC_009600), *Macadamia integrifolia* (NC_025288), *Megaleranthis saniculifolia* (NC_012615), *Meliosma* aff. *Cuneifolia* (NC_029430), *Nandina domestica* (NC_008336), *Nelumbo nucifera* (NC_025339), *Nicotiana tabacum* (NC_001879), *Nuphar advena* (NC_008788), *Oryza sativa* (NC_001320), *Pachysandra terminalis* (NC_029433), *Panax ginseng* (NC_006290), *Papavr somniferum* (NC_029434), *Platanus occidentalis* (NC_008335*)*, *Populus alba*(NC_008235)*, Ranunculus macranthus* (NC_008796)*, Sabia yunnanensis* (NC_029431), *Sinopodophyllum hexandrum* (NC_027732), *Stephania japonica* (NC_029432), *Thalictrum coreanum* (NC_026103), *Trochodendron aralioides* (NC_021426).
